# Fast multi-compartment Microstructure Fingerprinting in brain white matter

**DOI:** 10.3389/fnins.2024.1400499

**Published:** 2024-07-19

**Authors:** Quentin Dessain, Clément Fuchs, Benoît Macq, Gaëtan Rensonnet

**Affiliations:** ^1^Institute of Information and Communication Technologies, Electronics and Applied Mathematics (ICTEAM), UCLouvain, Louvain-la-Neuve, Belgium; ^2^Institute of NeuroScience, UCLouvain, Brussels, Belgium

**Keywords:** diffusion MRI, deep learning, microstructure, fingerprinting, non-negative linear least-squares, crossing bundles

## Abstract

We proposed two deep neural network based methods to accelerate the estimation of microstructural features of crossing fascicles in the white matter. Both methods focus on the acceleration of a multi-dictionary matching problem, which is at the heart of Microstructure Fingerprinting, an extension of Magnetic Resonance Fingerprinting to diffusion MRI. The first acceleration method uses efficient sparse optimization and a dedicated feed-forward neural network to circumvent the inherent combinatorial complexity of the fingerprinting estimation. The second acceleration method relies on a feed-forward neural network that uses a spherical harmonics representation of the DW-MRI signal as input. The first method exhibits a high interpretability while the second method achieves a greater speedup factor. The accuracy of the results and the speedup factors of several orders of magnitude obtained on *in vivo* brain data suggest the potential of our methods for a fast quantitative estimation of microstructural features in complex white matter configurations.

## 1 Introduction

Quantitative MRI (qMRI) aims to supply objective measurable metrics that specifically depict the morphology, microstructure, and/or chemical composition of tissues in order to provide a deeper knowledge of the physiology of the brain *in vivo* (Cercignani et al., 2018). For instance, Magnetic Resonance Fingerprinting (MRF) (Ma et al., 2013) pioneered an approach in which MRI data are acquired with time-varying acquisition parameters (e.g., flip angle, echo time, time of repetition) and then matched to a dictionary of pre-simulated fingerprints with identical parameters time course. Such a process determines the T1, T2, B0, and proton density parameters used to generate the fingerprint. This not only enables a very fast estimation of T1, T2, B0, and proton density maps, reduced to a simple dictionary matching, but also allows the use of the Bloch Simulator (Doneva et al., 2010), which is one of the most accurate ways to model the physics of MRI. The MRF model has then been extended to account for more complex tissue parameters such as B1+, T2*, perfusion (Su et al., 2017; Wright et al., 2018), hemodynamic (Christen et al., 2014; Lemasson et al., 2016), and diffusion (Cao et al., 2024) related properties. However, this requires the use of ever growing dictionaries which renders the dictionary matching step of MRF very time consuming due to the considerable amount of fingerprints required.

Subsequent advancements demonstrated the feasibility of extracting morphological characteristics of brain tissues using diffusion-weighted magnetic resonance imaging (DW-MRI) by matching the observed DW-MRI signal to a simulated signal counterpart mirroring predefined morphological features (Rensonnet et al., 2015; Palombo et al., 2016). This approach inspired Microstructure Fingerprinting (MF) (Rensonnet et al., 2019), which uses a precalculated dictionary to estimate microstructural properties of the white matter from DW-MRI. Instead of the Bloch equations, a Monte Carlo simulator solves the Bloch-Torrey equation to simulate diffusion fingerprints in a variety of white matter tissue configurations, flexibly incorporating information about the axonal density, diameter, principal orientation, their geometries, and sizes. MF was designed to deal with multi-compartment models from the beginning because of the nature of the white matter, composed of multiple populations of axons intersecting throughout the brain. When the fascicles overlap in a voxel, the model assumes that the total diffusion-weighted MRI signal is the sum of the signals originating from each fascicle independently (Rensonnet et al., 2018). This allows the use of multiple small dictionaries instead of a much larger one, accounting for all the compartment orientations and fractions, considerably decreasing the time and memory requirements by reducing the problem size. However, selecting the right combination of fingerprints remains combinatorial. The runtime complexity of MF is therefore affected by the number of fascicles in a voxel as $O\left(K\cdot N^{K}\right)$, where *N* represents the number of elements in the (single-fascicle) dictionary and *K* the number of fascicles in the voxel (Rensonnet et al., 2021). This severely limits the size and thus the complexity of the precalculated dictionary compared to the theoretically limitless possibilities of Monte Carlo simulation tools.

Kiselev et al. (2021) highlighted the importance of physiology-based models and the potential synergy between microstructure MRI and magnetic resonance fingerprinting for achieving specific, target-oriented diagnostic tools. Aligning with these recommendations, other advanced models similar to MF have been developed. For instance, Filipiak et al. (2022) employed a dictionary of presimulated Orientation Distribution Functions based on a biophysically plausible multicompartment diffusion model to accurately estimate fiber crossings with shallow angles.

In both MRF (always dictionary-based) and DW-MRI estimation (either dictionary-based or not), deep neural networks (DNN) are increasingly used to accelerate estimations of complex tissue models. Indeed, in order to find hierarchical representations that can handle challenging tasks with large-scale datasets, DNNs have proven to be a very effective tool (Najafabadi et al., 2015). As illustrated in Table 1, a number of studies have proposed deep learning methods to bypass long non-linear models fitting used in dMRI and to circumvent lookup in dictionaries of ever-increasing sizes in MRF.

**Table 1:** Fast estimation of tissue parameters from magnetic resonance measurements has relied on a variety of machine and deep learning approaches, usually assuming one main population of axons.

	**Trained on synthetic data**	**Trained on real data**	**Neural Network architecture**	**Type of data simulator**	**Type of acquisition protocol**	**# of axonal populations per voxel**
Cai et al. (2018)	✓	✗	CNN	Bloch equations	MRF	
Nedjati-Gilani et al. (2017)	✓	✗	Random Forest regression	Monte Carlo simulator	dMRI	1
de Almeida Martins et al. (2021)	✓	✗	MLP	Standard model of diffusion with relaxation	dMRI	1
Sabidussi et al. (2021)	✓	✗	Recurrent inference machines	Model based generation framework with gaussian noise	CINE	1
Karimi et al. (2021)	✓	✓	MLP with ReLU	Based on a multi tensor model	dMRI	1
Hill et al. (2018)	✓	✗	MLP	Monte Carlo simulator	dMRI	1
Ye et al. (2019)	✗	✓	Two-stage architecture using a latent space	Not Applicable	dMRI	1
*Proposed Hybrid Method*	✓	✗	Two-stage architecture using a latent space	Monte Carlo simulator	dMRI	2
*Proposed Fully-Learned Method*	✓	✗	Spherical harmonics decomposition + MLP	Monte Carlo simulator	dMRI	2

Comparison of state-of-the-art methods.

Simulated data have been used for training (Nedjati-Gilani et al., 2017; Cai et al., 2018; de Almeida Martins et al., 2021; Karimi et al., 2021; Sabidussi et al., 2021) because they allow any combination of parameter values and facilitate the creation of substantial datasets. Moreover, simulated data offer a ground truth which is not available using real data. When generating the synthetic dataset, de Almeida Martins et al. (2021), Karimi et al. (2021), and Sabidussi et al. (2021) opted for a closed form model based on several assumptions, while Cai et al. (2018) used the Bloch equations to numerically generate simulated data to train convolutional neural networks (CNN) (LeCun et al., 1998) used for T1 and T2 mapping. By using a numerical simulator instead of a closed-form model, an enhanced biophysical accuracy is obtained at the cost of an increased use of computational resources.

Ye (2017) and Ye et al. (2019) proposed a two-stages method for the acceleration of the estimation of the NODDI parameters (Zhang et al., 2012). The first stage is similar to a solution to a dictionary-based sparse reconstruction problem while the second stage computes the final NODDI microstructure estimation. They jointly learn the weights of the two stages by minimizing the mean squared error (MSE) of microstructure estimation. The separation of the problem into multiple steps instead of using an end-to-end DNN allows them to reduce the complexity of the problem and accelerate training.

In this paper, we propose two methods aimed at accelerating DW-MRI microstructure estimation using DNN. The first method, the “Hybrid Method,” exploits the power of presimulated Monte Carlo dictionaries by projecting the measured DW-MRI signal *y* into an interpretable latent space, representing the signal in terms of dictionary fingerprints, before providing it to a feed-forward neural network. This allows for generalization to different acquisition schemes with little to no additional training. The second method, the “Fully-Learned Method,” is based on a feed-forward neural network that uses the spherical harmonics representation of the DW-MRI signal as input to jointly estimate microstructural features and fascicles orientations. This method is less interpretable than the Hybrid Method but is significantly faster since no minimization step is required.

We evaluated each method in terms of the inference speed and accuracy of their fittings on synthetic and *in vivo* datasets. First, we estimated the accuracy and noise robustness via experiments on simulated data with varying noise levels. Second, we performed a second accuracy and noise robustness assessment on the Hybrid Method with data generated from an unseen acquisition protocol to evaluate its generalizability. Finally, we tested the quality of each method to retain small structures within the brain, using 34 *in vivo* scans from the MGH-USC Young Adult cohort (Van Essen et al., 2012).

## 2 Materials and methods

This section begins with a detailed presentation of the reference methodology, known as Microstructure Fingerprinting (MF). We then introduce and elaborate on the two proposed acceleration techniques: the Hybrid Method and the Fully-Learned Method. Subsequently, the section delineates the three experiments carried out to evaluate and compare these three methods, focusing on assessing their inference speed and mean absolute error (MAE). This comparative analysis aims to provide a comprehensive understanding of the performance and potential advantages of each method within the context of DW-MRI microstructure estimation.

### 2.1 Microstructure Fingerprinting

**Figure 1:**
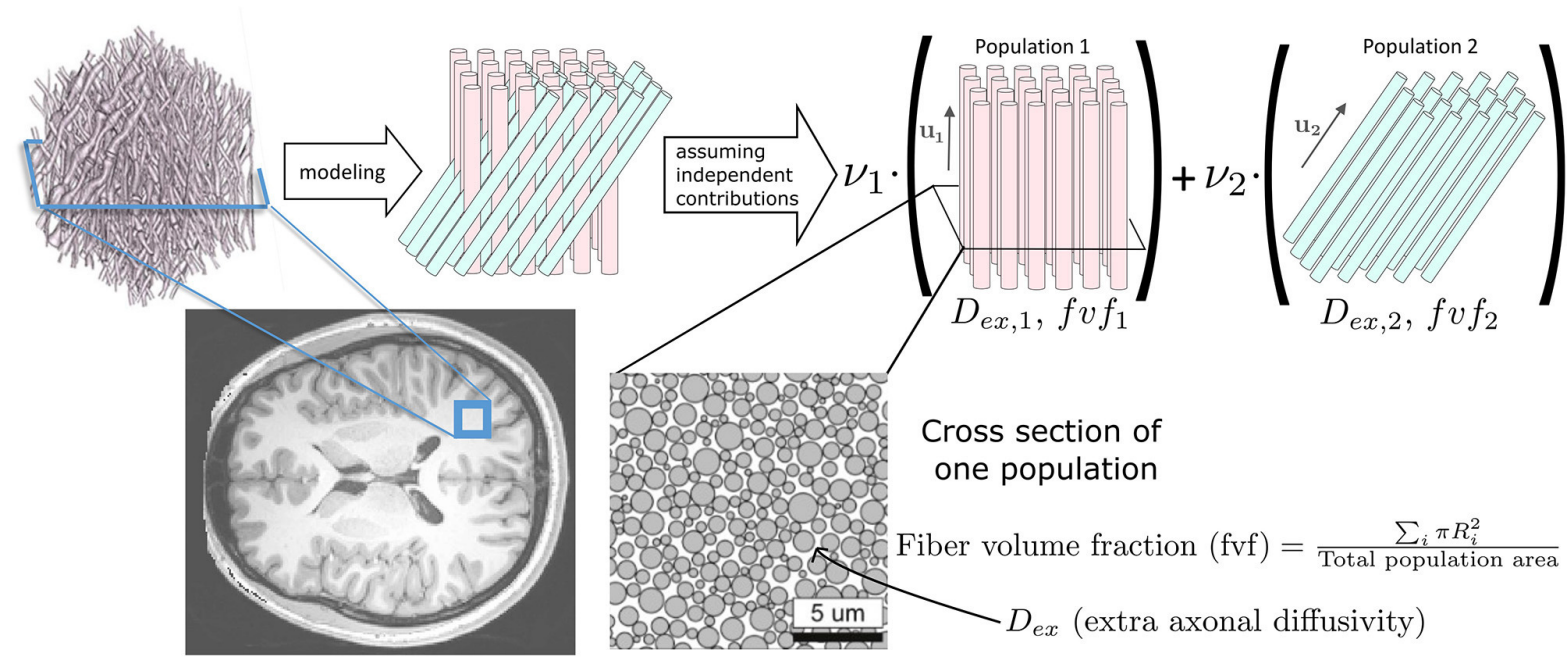
Our forward signal model exploits Monte Carlo numerical simulations. A voxel of white matter [artistic view borrowed from Ginsburger et al. (2018)], located here on a T1 anatomical scan of a healthy young adult from the Human Connectome Project (Van Essen et al., 2012), is represented by axon populations modeled as straight cylinders with signal contributions assumed independent. The DW-MRI signal is obtained by Monte Carlo simulations in each population (Hall and Alexander, 2009; Rensonnet et al., 2015).

The Microstructure Fingerprinting framework first precomputes a dictionary of DW-MRI fingerprints (Rensonnet et al., 2015, 2019). Each fingerprint in the dictionary corresponds to a unique microstructural configuration, obtained by leveraging the physical accuracy of Monte Carlo simulations (Hall and Alexander, 2009; Rensonnet et al., 2015). As depicted in Figure 1, in this work, every axonal configuration was a random 2D packing of straight cylinders with radii drawn from a gamma distribution with mean radius of 0.5 μm and a standard deviation of 0.3 μm. The intra-axonal diffusivity was set to 2.2 μm2 ms-1 (Dhital et al., 2019). The different configurations present in the dictionary were obtained by choosing the fiber volume fraction (*fvf*) from 38 equally spaced values within the range [0.06, 0.8] and the extra-axonal diffusivity (*D*_*ex*_) from 10 equally spaced values in the [0.6, 2.4] μm2 ms-1 range, totaling *N* = 380 precomputed fingerprints.

**Figure 2:**
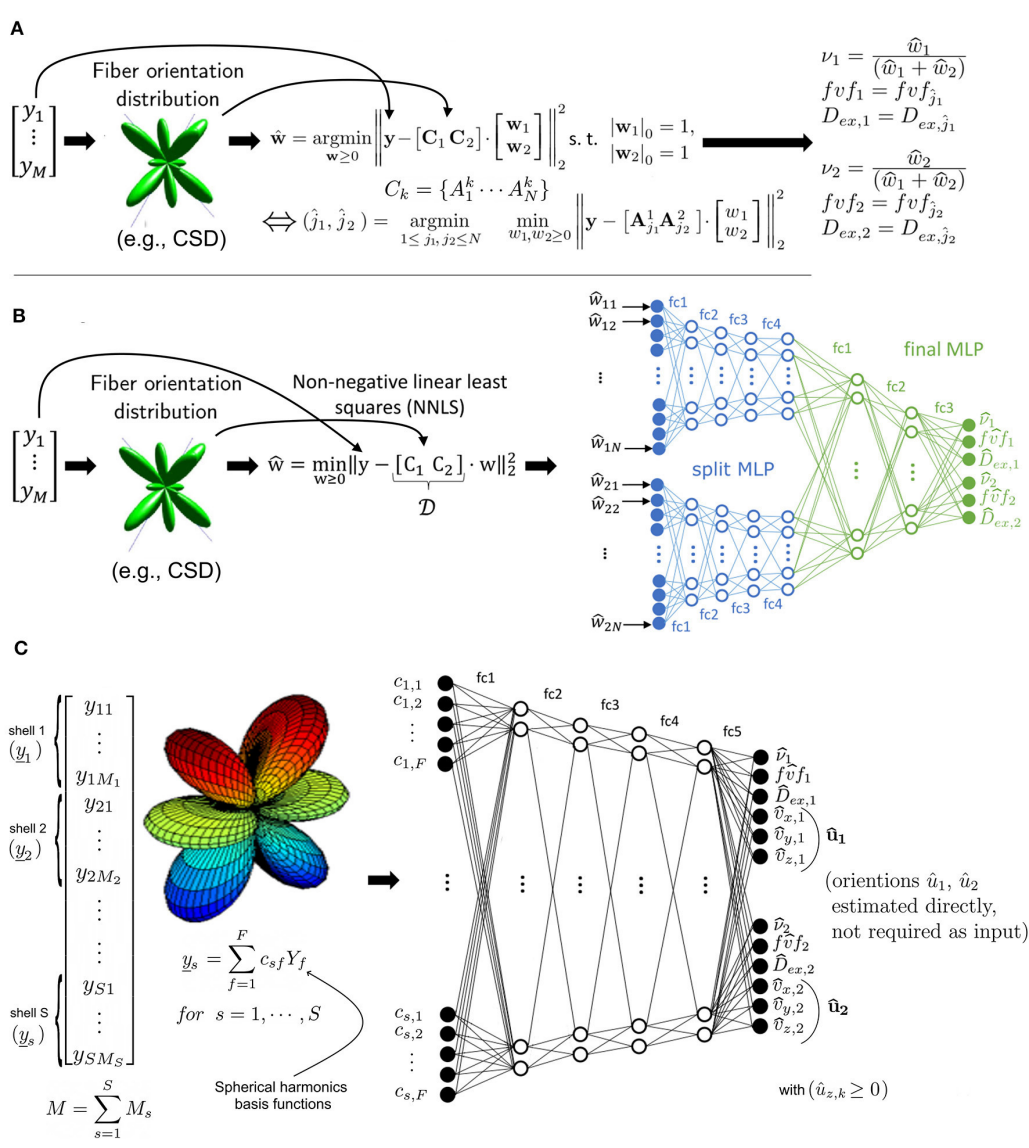
**(A)** Microstructure Fingerprinting involves solving a large number of small NNLS problems, leading to long computation times as the dictionary size N increases. **(B)** In the Hybrid Method, the vector of measurements $\hat{y}$ is decomposed by NNLS into a sparse representation in the space of physics-based fingerprints. The weights are given to a multilayer perceptron (MLP) with a split architecture to predict the tissue parameters (relative volume of axon population ν_*k*_, fiber volume fraction *fvf*_*k*_ and extra-axonal diffusivity *D*_*ex, k*_). **(C)** The Fully-Learned Method calculates shellwise spherical harmonics coefficients of order 12, which are fed into a DNN to predict the tissue parameters and axon populations orientation.

As shown in Figure 2A, at every runtime, MF requires an estimate of the orientations **u**_*k*_ of each fascicle *k* in every voxel. For this purpose, we here employed a constrained spherical deconvolution (CSD) (Jeurissen et al., 2014). Assuming independent signal contributions from each fascicle (Rensonnet et al., 2018), the precomputed dictionary was then rotated to create fascicle-specific sub-dictionaries **C**^*k*^. The method then searched for the optimal combination of single-fascicle configurations (*fvf*_*k*_, *D*_*ex, k*_) and volume fractions ν_*k*_, for each fascicle *k* = 1, …, *K*, given a vector **y**∈ℝ^*M*^ of *M* noisy DW-MRIs by selecting the best-fit solution out of *N*^*K*^ independent non-negative linear least-squares (NNLS) sub-problems of *K* variables.

### 2.2 The Hybrid Method

The Hybrid Method operates in two distinct stages. In the first stage (Figure 2B), a relaxed version of the problem present in MF was used to project the DW-MRI signal into a latent space. Specifically, the same matrix [*C*_1_…*C*_*K*_] was first assembled. However, instead of tackling the problem with its full multi-compartment complexity, one single NNLS problem with *K*·*N* variables was solved without any knowledge about its multi-compartment structure and without any sparsity constraints on the weight vector **w**. This effectively projected the measurements **y** in the space of the dictionary fingerprints. NNLS is known to naturally enforce sparsity (Lawson and Hanson, 1995; Canales-Rodŕıguez et al., 2019). Additionally, its empirical runtime is proportional to the number of matrix columns, which in this case is *K*·*N*. This characteristic of NNLS is a key factor in the efficiency of the Hybrid Method.

As illustrated in Figure 2B, the second stage consisted in passing the NNLS weight vector **w** through a feed-forward neural network. The network initially had split arms, independently processing the weights related to each compartment by NNLS before merging into a common multi-layer perceptron (MLP) for final parameter inference. The advantage of this architecture is to reduce the number of free parameters and facilitate training. The output size of the two initial branches of the MLP, the learning rate and the number of layers were determined through a systematic hyperparameter optimization process using grid search. Our network used a rectified linear unit (ReLU) (Nair and Hinton, 2010) as an activation function for all layers, except for the last layer, where a sigmoid function was used. Between all layers, dropout regularization (Hinton et al., 2012) was used with a retention probability of 0.9. The network included a batch normalization layer (Ioffe and Szegedy, 2015) between the the split arms and common multi-layer perceptron. The Adam optimizer (Kingma and Ba, 2014) was utilized during training. The target output of the MLP for each fascicle *k* was the fascicle fraction ν, the fiber volume fraction *fvf* and the extra-axonal diffusivity *D*_*ex*_. For more in-depth details on models architecture and parameters used during training, see Supplementary Table S1.

The network was trained on a dataset comprising 20,00,000 synthetic voxels. These voxels were generated using Monte Carlo simulations based on the microstructural configuration presented in Figure 1, considering 2 fascicles per voxel. Microstructural parameters were drawn from uniform distributions: the crossing angle between the two axon populations ${\hat{\text{u}}}_{1}$ and ${\hat{\text{u}}}_{2}$ from $U\left(\left[15^{•},90^{•}\right]\right)$ and the volume fractions ν from U([0.05,0.95]). For each voxel, the pairs (*fvf*_*k*_, *D*_*ex, k*_) were randomly selected from the *N* = 380 precomputed fingerprints described above. The resulting DW-MRI signal *s* was corrupted with Rician noise using an i.i.d. Gaussian realization ϵ with standard deviation $\sigma =\frac{s_{\text{noiseless}}\left(\text{b-value}=0\right)}{\text{SNR}}$ by computing $s=\sqrt{s_{\text{noiseless}}^{2}+\epsilon^{2}}$. The signal-to-noise ratio (SNR) was drawn from U([10,100]) .

### 2.3 The Fully-Learned Method

As depicted in Figure 2C, the Fully-Learned Method relies on a traditional MLP architecture and directly outputs estimates of microstructural parameters without resorting to any intermediate biophysical representation of the signal. The Fully-Learned Method requires the DW-MRI signal to be acquired in shells (Tuch et al., 2002) of fixed b-value as it first computes a spherical harmonics representation of each shell separately to provide the coefficients as inputs to the network. Compared to the use of raw DW-MRI signal directly, this approach is more flexible with respect to changes in the acquisition protocol (e.g., in the number of gradients in some shells), and robust to missing measurements.

An important difference with the Hybrid Method is that we do not need to give the orientations **û**_*k*_ because they are estimated from the data jointly with the other fascicle-specific properties. Consequently, the approach does not require external estimates such as those of CSD. Note that, given the spherical symmetry of the DW-MRI signal, the orientations were restricted to the unit half-sphere $\left\{{\left[{û}_{x},{û}_{y},{û}_{z}\right]}^{T}|{û}_{z}\geq 0\right\}$ during training.

As in the Hybrid Method, a grid search approach was employed for the optimization of the neural network hyperparameters. ReLU and dropout were incorporated into each layer, except for the final layer, which utilized a sigmoid as the activation function and did not include dropout. The Adam optimizer was used during training. As we used real-valued of even-degree spherical harmonics functions up to degree 12, the input contained 91 coefficients per shell.

The training of the Fully-Learned Method was conducted using the same set of simulated data as for the Hybrid Method, ensuring consistency in the evaluation of both methods.

### 2.4 Diffusion protocol

Unless otherwise specified in the experiments below, the acquisition protocol used to simulate the DW-MRI samples was the one from the MGH Human Connectome Project (HCP) (Setsompop et al., 2013), consisting of 4 PGSE shells of 64 directions at *b* = 1,000 (*G* = 69 mT m-1), 64 at *b* = 3,000 (*G* = 120 mT m-1), 128 at *b* = 5,000 (*G* = 155 mT m-1) and 256 at *b* = 10,000µ*s* µ*m*-2 (*G* = 219 mT m-1) with gradient duration δ = 12.9 ms, diffusion time Δ = 21.8 ms, TE/TR=57/8800 ms and no MultiBand. A non-diffusion weighted image (b = 0) was collected every 14 volumes, yielding a total of 552 volumes. The total acquisition time was 89 min.

### 2.5 Experiment I: performance assessment on synthetic test set

#### 2.5.1 Two-fascicle configuration

Based on the diffusion protocol used during training, a test set was created with 2-fascicle voxels having crossing angles drawn from $U\left(\left[15^{•},90^{•}\right]\right)$, ν_1_ values selected in the set [0.5, 0.6, 0.7, 0.8, 0.9], every (*fvf*_*k*_, *D*_*ex, k*_) pair from the *N* = 380 precomputed fingerprints described above and SNR values in the set [20, 30, 50], generating a total of 150,000 samples. All testing samples were never seen by either neural networks during training.

The experiment focused on assessing four distinct models. The first two are the proposed accelerated methods, known as the Hybrid Method and the Fully-Learned Method. Alongside these, two versions of the Microstructure Fingerprinting (MF) model were assessed. The True orientations & MF, which utilizes actual, known fascicle orientations, serving as a benchmark for the ideal scenario in Microstructure Fingerprinting. In contrast, the CSD & MF model operates without the advantage of known fascicle orientations and instead relies on orientations estimated through constrained spherical deconvolution, depicting a scenario more representative of typical clinical settings.

For each of these models, the absolute error and mean absolute error were calculated on the estimated volume fractions ν_1_ and ν_2_, extra-axonal diffusivities *D*_*ex*, 1_ and *D*_*ex*, 2_, fiber volume fractions *fvf*_1_ and *fvf*_2_. We also reported the mean angular error on û_1_ and û_2_, computed either by CSD or by the Fully-Learned Method directly.

#### 2.5.2 Three-fascicle configuration

To evaluate the performance of our models in more complex scenarios involving three-way fascicle crossings, the architectures of both the Hybrid and Fully-Learned neural networks were modified to process three-fascicle voxels. These models were retrained on synthetic data employing similar configurations to those used in the two-fascicle experiments, with an alteration in the method of volume fraction generation. Given the difficulty of using an uniform distribution for ν_*k*_ in scenarios involving more than two fascicles while ensuring $\underset{k}{\sum }\nu_{k}=1$, volume fractions ν_*k*_ were generated using a Dirichlet distribution with α = 1.

For the test set, the methodology from the two-fascicle experiment was maintained, but the uniform distribution for volume fractions was replaced with a Dirichlet distribution. Following the procedures established in the two-fascicle tests, all four methods were evaluated.

### 2.6 Experiment II: generalizability to unseen acquisition protocols

The objective of this experiment was to evaluate the Hybrid Method's ability to generalize to new, previously unseen DW-MRI acquisition protocols. This was driven by the fact that, unlike the Fully-Learned Method, the Hybrid Method represents the DW-MRI signal by an intermediate weight vector **w** that does not depend on the number of measurements *M* but only on the underlying microstructural model via the dictionary of fingerprints.

For this experiment, a new simulated protocol was designed to mimic current clinical capabilities in DW-MRI with gradient duration δ = 22.9 ms, diffusion time Δ = 35.7 ms, TE/TR=77.4/4842 ms, 64 gradient directions at *b* = 1,000 (*G* = 31 mT m-1) and *b* = 2,000 (*G* = 44 mT m-1), and 128 directions at *b* = 5,000 µ*s* µ*m*-2 (*G* = 69 mT m-1), along with 4 *b* = 0 acquisitions, for a total of 260 measurements. The simulated protocols excluded gradient directions at *b* = 10,000 µ*s* µ*m*-2 since high b-values are typically impractical in clinical settings. Furthermore, to create a clear distinction from the reference protocol used during training, gradient directions at *b* = 3,000 µ*s* µ*m*-2 were also omitted.

The test set for this experiment was generated following the same methodology as in Experiment I, but adapted to the new protocol. In this experiment, four distinct methods were put to the test: The MF model with true fascicle orientations (True orientations & MF) and without true fascicle orientations (CSD & MF) as in Experiment I, and the Hybrid Method under two different conditions. The first condition involved applying the Hybrid Method without any retraining, directly to the new protocol. This gauges the method's inherent ability to adapt to different acquisition parameters based on its original training. The second condition involved fully retraining the Hybrid Method specifically for the new protocol, aiming to determine the advantages of tailoring the model to the unique characteristics of a new protocol.

### 2.7 Experiment III: *in vivo* population

Experiment III focuses on estimating the microstructural properties of white matter on *in vivo* data for the CSD & MF, Hybrid Method, and Fully-Learned Method. This assessment was performed on all 34 subjects from the MGH-USC Young Adult cohort (Van Essen et al., 2012), whose ages ranged from 20 to 59 years old and scanned using a customized Siemens 3T Connectome scanner, providing a consistent and high-quality dataset for analysis. The computation time required by each model to process the MRI data from all 34 subjects was recorded to assess inference speed. The white matter maps generated by the two accelerated methods were compared with those produced by the reference MF method across all subjects and a visual inspection was performed. Given the lack of ground truth on *in vivo* data, the experiment also aimed to discern the effects of transitioning from synthetic to real-world data. To achieve this, the differences in the estimated fiber volume fraction (*fvf*_1_) between the reference MF and the two proposed methods were calculated for each voxel. These calculations were performed on both the simulated test set from Experiment I and the *in vivo* data from the 34 subjects in the cohort. The results were presented as histograms of the signed differences, providing a statistical perspective on the variance between each method's estimations when applied to synthetic and real-world data.

## 3 Results

### 3.1 Experiment I: performance assessment on synthetic test set

#### 3.1.1 Two-fascicle configuration

Experiment I evaluated the accuracy and efficiency of our proposed methods, the Hybrid Method and the Fully-Learned Method, against conventional Microstructure Fingerprinting (MF) approaches, using a synthetic test set designed to simulate diverse white matter microstructures.

As visible in Table 2, both methods yielded the expected gain in computation time with the Hybrid Method reaching an acceleration factor of 38.9 and the Fully-Learned Method reaching an even higher acceleration factor of 2, 090. A more detailed view of computation time is available in Supplementary Table S2.

**Table 2:** Large speed-up factors were observed for our proposed methods, with theoretical projections suggesting even larger gains with increasing dictionary size (N) and number of compartments (K).

	**Microstructure Fingerprinting**	**Hybrid Method**	**Fully-Learned Method**
Precomputation time	≈2 d	≈2 d	Not applicable
**Case with K = 2 fascicles**			
Model Training	Not applicable	7 min	21 min
Total inference time/voxel	9.18 × 10 − 1 s	2.36 × 10 − 2 s	8.61 × 10 − 4 s
**Acceleration factor**	**1**	**38.9**	**2090**
**Case with K = 3 fascicles**			
Model Training	Not applicable	10 min	22 min
Total inference time/voxel	248 s	2.97 × 10 − 2 s	8.82 × 10 − 4 s
**Acceleration factor**	**1**	**8350**	**281179**
Inference complexity	$O\left(N^{K}K\right)$	O(NK)	O(1)
Theoretical acceleration factor	O(1)	$O\left(N^{K-1}\right)$	$O\left(N^{K}K\right)$

Efficiency of the three methods when inference is performed on synthetic data using the MGH Adult Diffusion protocol. The test set was processed on a Skylake Xeon 4116 CPU cluster, without GPU acceleration. During the training phase, a Nvidia RTX 3090 GPU was used to train both proposed methods. The bold values are used to emphasize the key information, specifically the acceleration factor.

**Figure 3:**
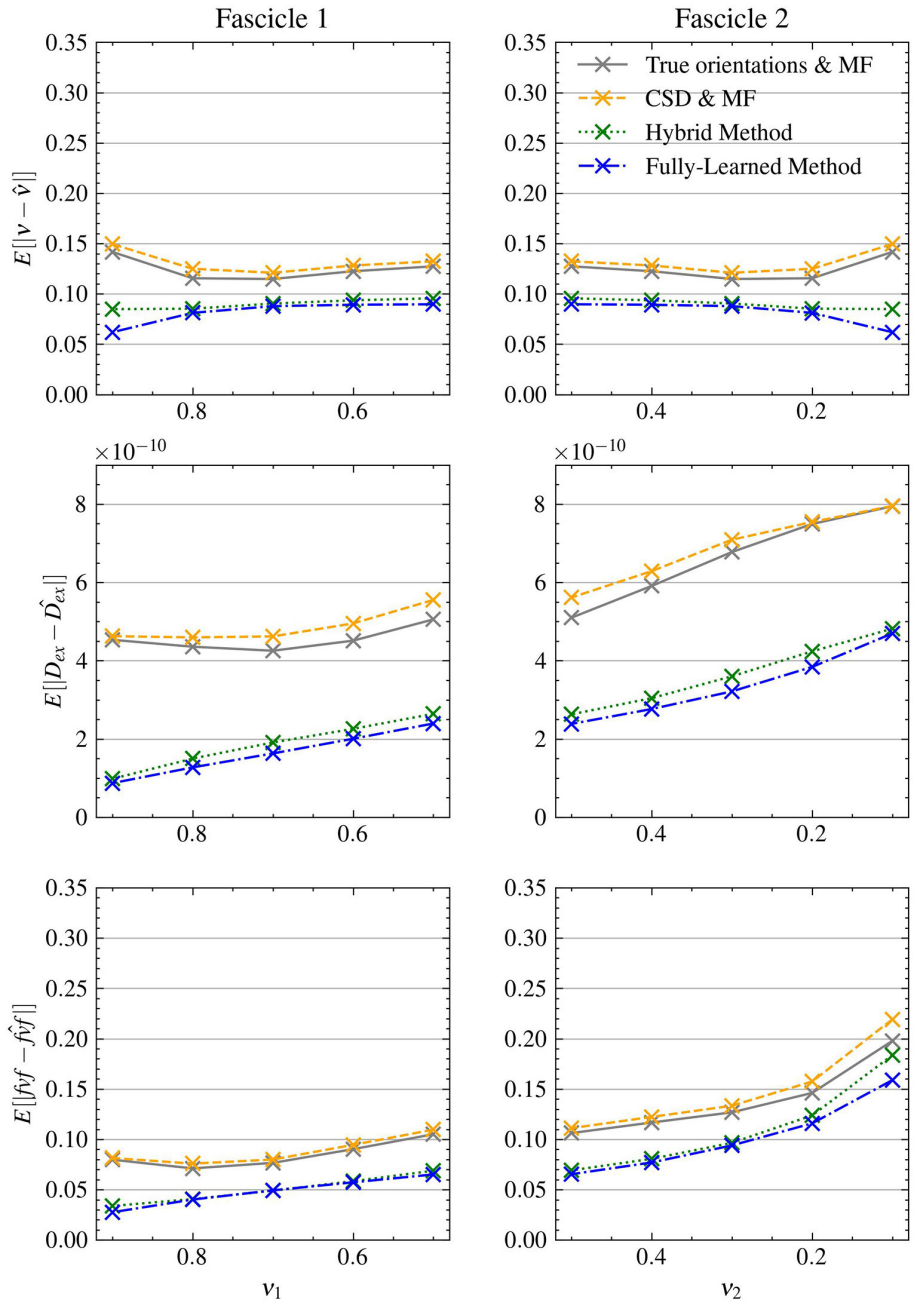
The Fully-Learned Method has better estimation accuracy than the Hybrid Method, the CSD & MF and the MF algorithm with true orientations. MAE on structured test.

Figure 3 presents the mean absolute errors (MAEs) by fascicle fraction ν for each microstructural feature estimated by the four evaluated models. Furthermore, Table 3 complements this by providing a detailed breakdown of the MAEs for each of the three signal-to-noise ratio (SNR) levels within the synthetic test set, thus offering insights into each method's accuracy under different noise conditions. The Hybrid Method yielded MAEs of 0.0899 for ν, 0.0805 for *fvf*, and 2.76e − 10 for *D*_*ex*_. The Fully-Learned Method demonstrated marginally better performance, with MAEs of 0.0819 for ν, 0.0751 for *fvf*, and 2.51e − 10 for *D*_*ex*_. In contrast, the True Orientations & MF method, despite utilizing accurate fascicle orientations, reported higher MAEs of 0.124 for ν, 0.112 for *fvf*, and 5.59e − 10 for *D*_*ex*_. The CSD & MF method marked the highest MAEs, with 0.131 for ν, 0.118 for *fvf*, and 5.88e − 10 for *D*_*ex*_. These results underscore the superior accuracy of both proposed methods over the traditional MF approach in estimating key microstructural features.

**Table 3:** Proposed acceleration methods outperform reference approaches.

	**SNR = 20**			**SNR = 30**			**SNR = 50**		
	**ν**	***D*_*ex*_**	***fvf***	**ν**	***D*_*ex*_**	***fvf***	**ν**	***D*_*ex*_**	***fvf***
True orientations & MF	0.149	6.77e − 10	0.123	0.125	5.65e − 10	0.114	0.100	4.37e − 10	0.098
CSD & MF	0.152	7.02e − 10	0.129	0.131	5.82e − 10	0.119	0.111	4.82e − 10	0.109
Hybrid Method	0.107	3.21e − 10	0.094	0.092	2.84e − 10	0.083	0.071	2.24e − 10	0.064
Fully-Learned Method	0.095	2.92e − 10	0.088	0.084	2.55e − 10	0.076	0.068	2.06e − 10	0.061

MAEs of all methods on the test set from Experiment 1. For each metric and SNR, the method with the lowest MAE is highlighted in bold.

**Figure 4:**
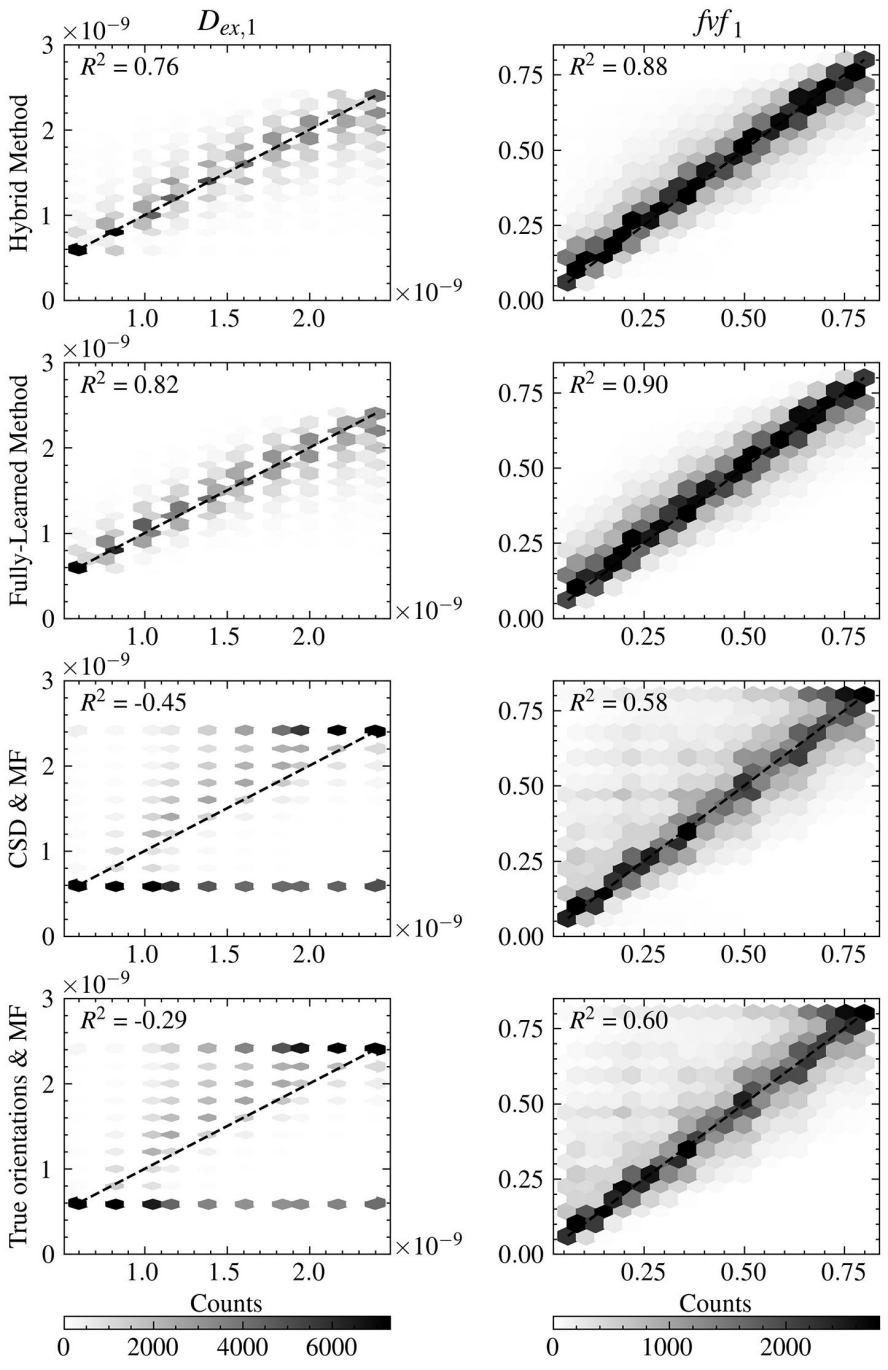
Both accelerated method obtain estimates closer to the groundtruth for the *fvf* and *D*_*ex*_ metrics than the reference MF methods. Correlation-accuracy plots comparing the predicted values and the ground truth parameters for the four models on the test set.

Further insight into the models' performance is provided by Figure 4, which includes correlation-accuracy plots for the *fvf*_1_ and *D*_*ex*, 1_ properties. This figure also reveals the coefficient of determination (*R*^2^), showcasing superior predictive accuracy for the Hybrid (0.88 for *fvf*_1_ and 0.76 for *D*_*ex*, 1_) and Fully-Learned Methods (0.90 for *fvf*_1_ and 0.82 for *D*_*ex*, 1_), in stark contrast to the lower *R*^2^ values observed for the CSD & MF (0.58 for *fvf*_1_ and −0.45 for *D*_*ex*, 1_) and True Orientations & MF methods (0.60 for *fvf*_1_ and −0.29 for *D*_*ex*, 1_).

**Figure 5:**
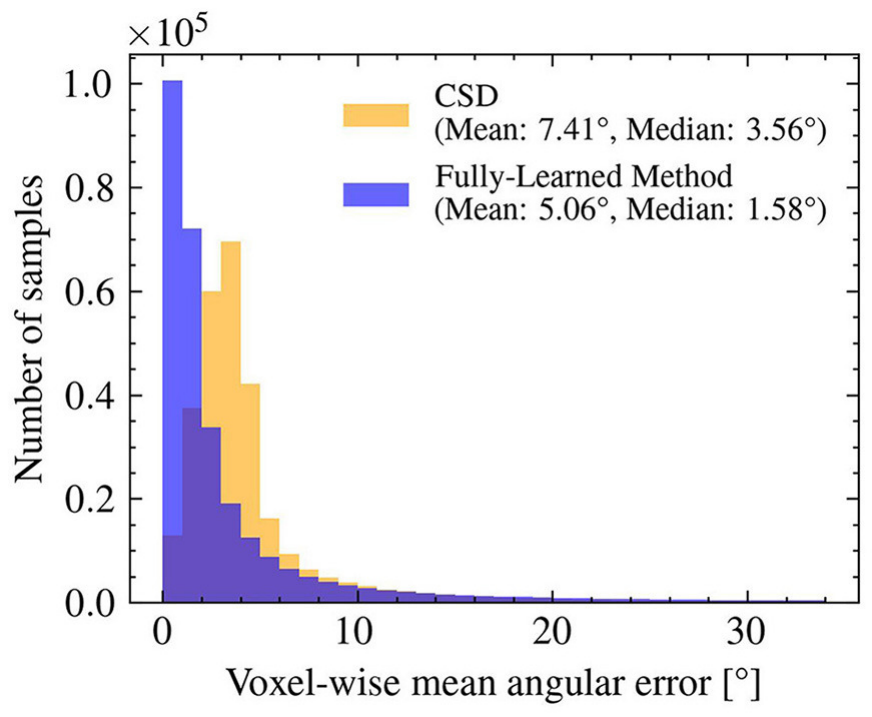
The orientations obtained with the Fully-Learned Method outperform results from constrained spherical deconvolution in the synthetic test set. Angular Error: CSD vs. the Fully-Learned Method.

As shown in Figure 5, the angular errors on the estimated orientations obtained by the Fully-Learned Method were smaller (mean = 5.06°) than those of CSD (mean = 7.41°), highlighting the capacity of the method to successfully estimate orientations.

#### 3.1.2 Three-fascicle configuration

To further assess the performance and scalability of the proposed methods, the second part of Experiment I evaluated all methods on voxels with a three-fascicle configuration. This extension aimed to test the robustness and efficiency of the methods under more complex scenarios.

As illustrated in Table 2, the acceleration factors achieved with the three-fascicle configuration are significantly higher than those observed with the two-fascicle configuration. Specifically, the Hybrid Method achieved an acceleration factor of 8,350, while the Fully-Learned Method reached 281,179.

**Figure 6:**
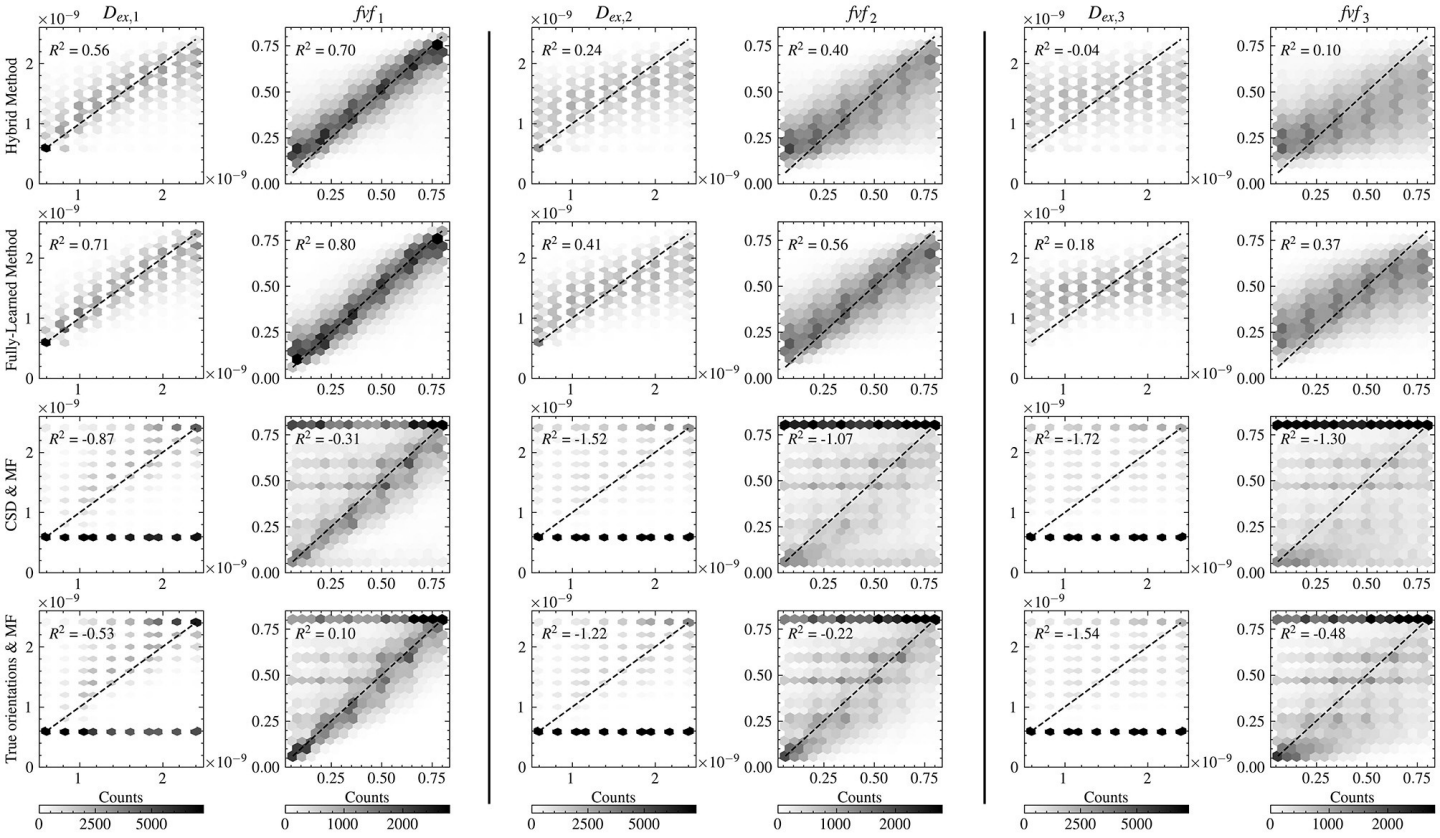
The accelerations methods generalize to three-way crossings, outperforming the reference MF methods in estimation accuracy for the metrics *fvf* and *D*_*ex*_. Correlation-accuracy plots comparing the predicted values against the ground truth parameters for the four models on the test set with a three-fiber configuration. The difference between actual and estimated values are shown for the first fascicle in the two left-most columns, for the second fascicle in the two middle columns, and for the third fascicle in the two right-most columns.

The correlation-accuracy plots in Figure 6 reveal the same trends as in the two-fascicle configuration, with acceleration methods obtaining estimates closer to the groundtruth than the reference MF methods. The errors are slightly higher overall in the three-fascicle configuration for all tested approaches.

### 3.2 Experiment II: generalizability to unseen acquisition protocols

**Figure 7:**
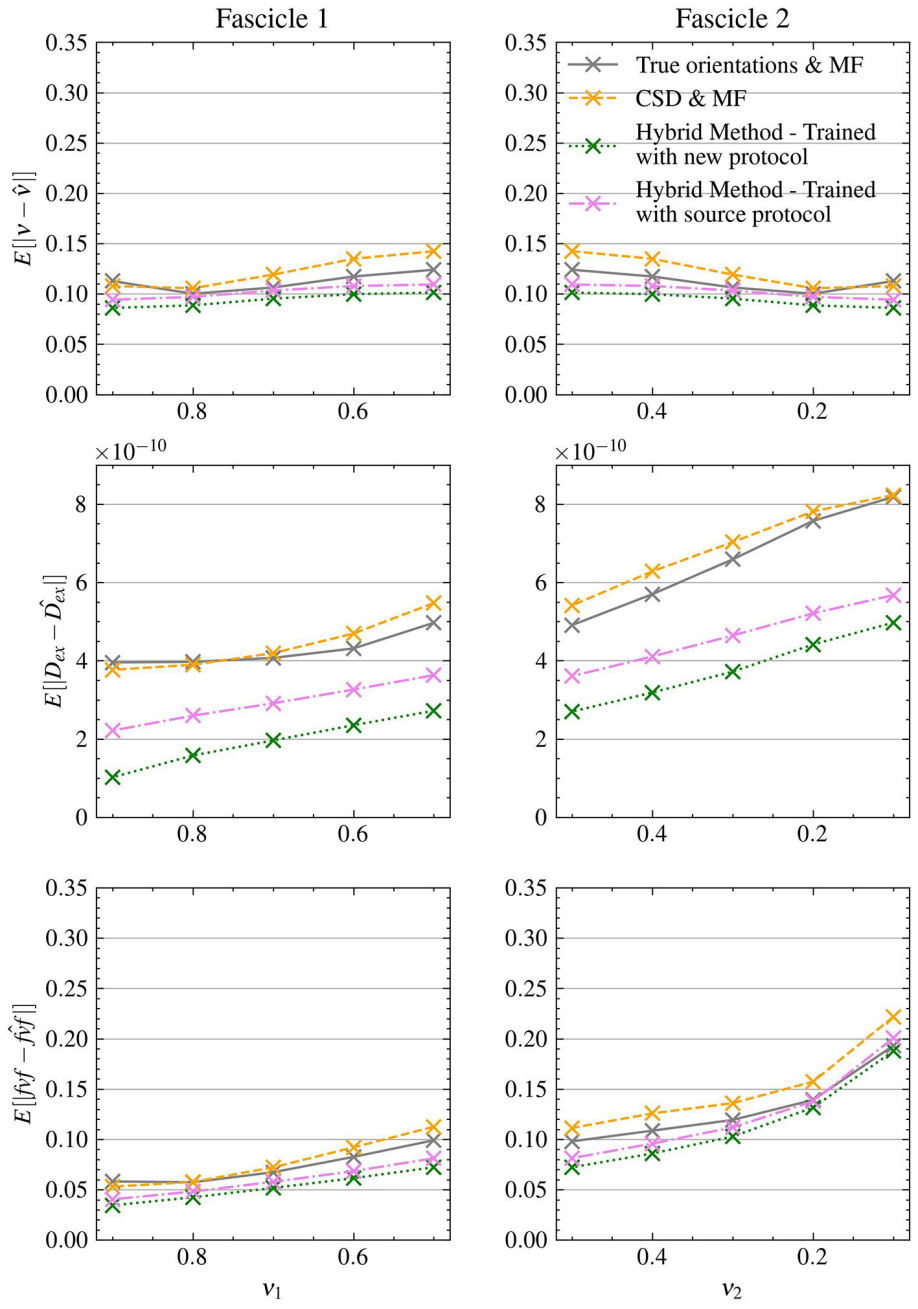
The Hybrid Method is able to generalize to data acquired with a different experimental protocol with minimal retraining. MAE on structured test with the clinical protocol.

The results from Experiment II, as depicted in Figure 7, demonstrate the ability of the Hybrid Method to transfer across different protocols. Without retraining, the Hybrid Method achieved MAEs of 0.102 for ν, 0.0923 for *fvf* and 3.79e − 10 for *D*_*ex*_, consistently outperforming the CSD & MF method in all scenarios which had MAEs of 0.122 for ν, 0.114 for *fvf* and 5.68e − 10 for *D*_*ex*_ along with the True orientations & MF approach which had MAEs of 0.112 for ν, 0.102 for *fvf* and 5.42e − 10 for *D*_*ex*_.

Upon retraining with the new protocol, the Hybrid Method's accuracy improved further, yielding even lower MAEs of 0.0942 for ν, 0.0843 for *fvf* and 2.86e − 10 for *D*_*ex*_, exhibiting an improvement in performance compared to own version trained on the source protocol. This enhanced performance was consistent across all metrics, further outstripping both reference methods.

### 3.3 Experiment III: *in vivo* population

Table 4 presents the acceleration factors achieved by these methods. The results were in line with the theoretical predictions regarding inference complexity.

**Table 4:** The acceleration factor obtained on *in vivo* data is of the same order of magnitude as the one obtained on synthetic data.

	**CSD & MF**	**Hybrid Method**	**Fully-Learned Method**
Computation of SH coefficients/cohort	Not applicable	Not applicable	47 min
Constrained spherical deconvolution	9.3 h	9.3 h	Not applicable
Dictionary rotation time/cohort	14.2 h	14.2 h	Not applicable
Exhaustive fingerprinting time/cohort	1690.5 h	Not applicable	Not applicable
Solving NNLS time/cohort	Not applicable	26.5 h	Not applicable
NN forward pass time/cohort	Not applicable	26 min	9 min
Total inference time/cohort	1714 h	50.4 h	56 min
Total inference time/subject	50.4 h	1.5 h	96 s
**Acceleration factor**	**1**	**34**	**1836**

Efficiency of the three methods when inference is performed on 34 subjects from the MGH Adult Diffusion dataset, processed on a Skylake Xeon 4116 CPU cluster with single-thread allocation per subject, without GPU acceleration. The bold values are used to emphasize the key information, specifically the acceleration factor.

**Figure 8:**
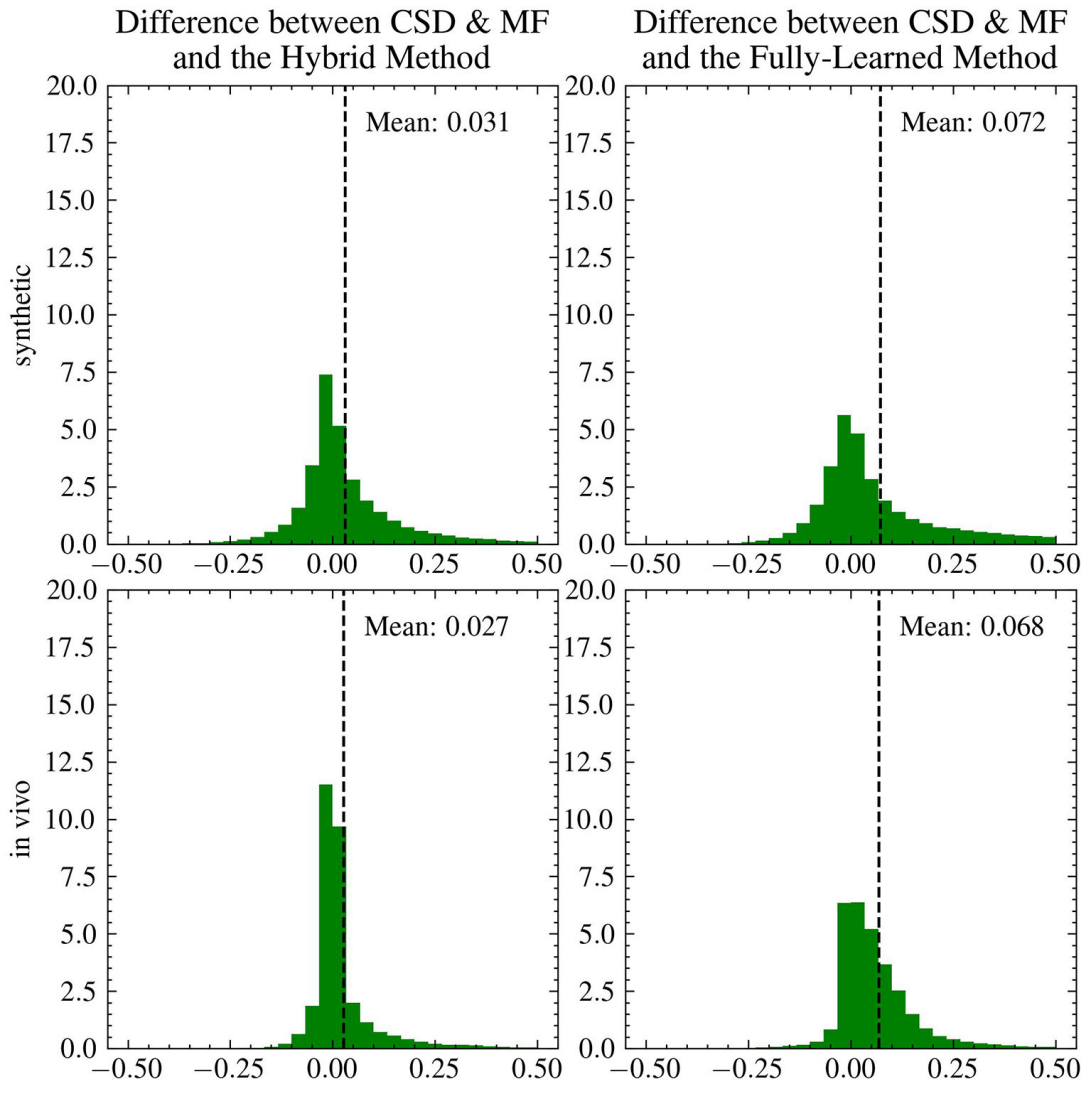
The mean differences between the reference and the accelerated Microstructure Fingerprinting follow similar trends on simulated and on *in vivo* data. Histogram (normalized to integrate to 1) of the signed differences between CSD & MF and the Hybrid **(left)** and Fully-Learned **(right)** Methods for *fvf*_1_ on synthetic **(top)** and *in vivo* **(bottom)** data. The vertical dotted lines represent the mean of the signed difference.

Figure 8 presents a comparative analysis between the CSD & MF method and each accelerated method, utilizing histograms to depict differences in *fvf*_1_ estimates across synthetic and *in vivo* datasets. Although the distribution of these differences changes slightly, the mean difference for both proposed methods maintains consistency as we transition from synthetic to *in vivo* data. Notably, the *in vivo* dataset shows a more tightly clustered signed difference between CSD & MF and the Hybrid Method around zero, indicating a closer alignment in real-world settings as opposed to synthetic environments. Meanwhile, the Fully-Learned Method consistently estimates higher fiber volume fraction values compared to the CSD & MF method.

**Figure 9:**
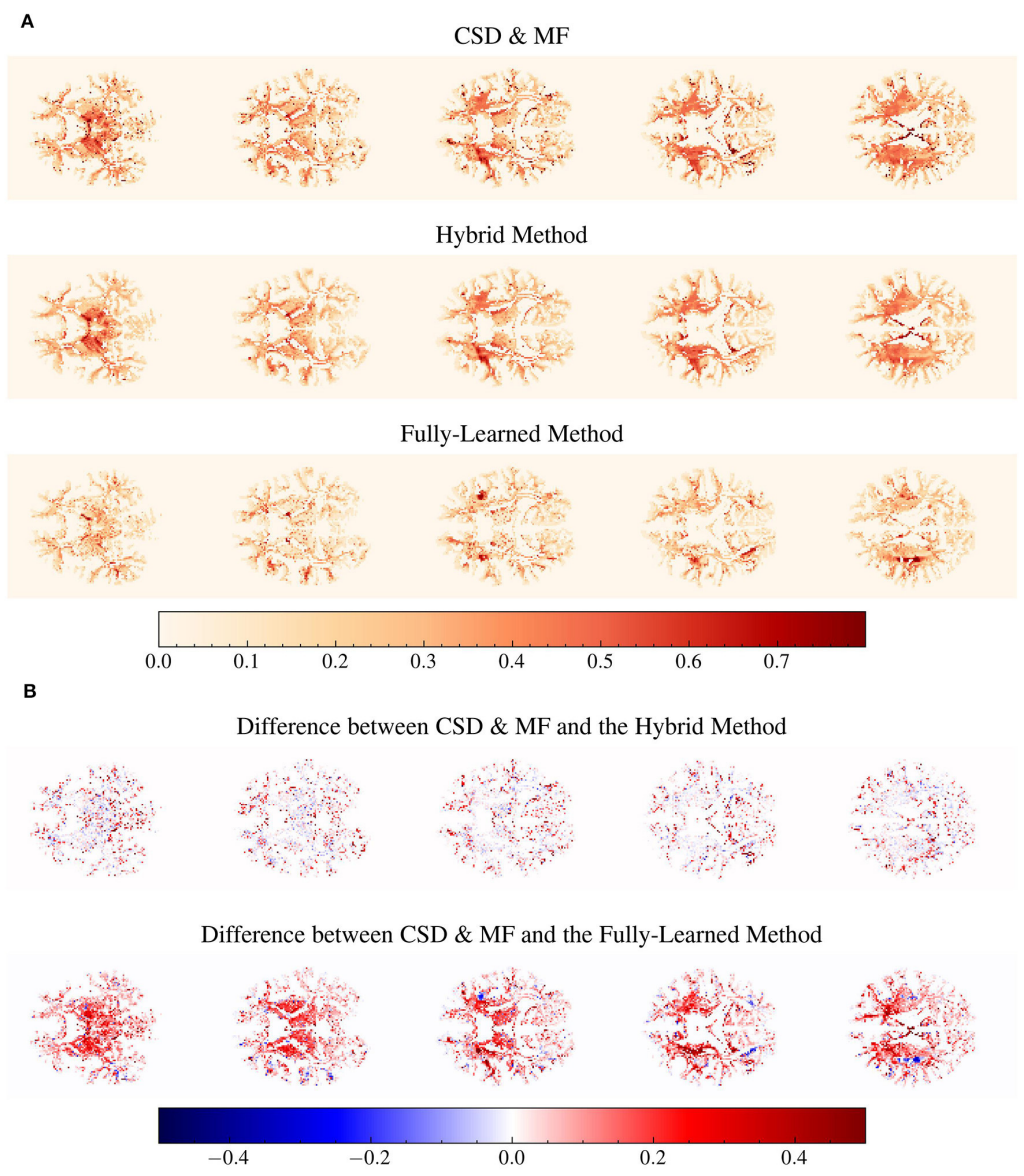
Structural integrity between all methods seems to be maintained. Several voxels are estimated to a higher fiber volume fraction by the Fully-Learned Method. Detailed visualization on subject MGH 1001 of voxel-wise maps of *fvf*_1_ and voxel-wise maps of the differences in fiber volume fraction for the first compartment (*fvf*_1_) between the reference method and the Hybrid Method and the Fully-Learned Method. Constrained spherical deconvolution (CSD) was used to estimate the number of fascicles and their orientations. The analysis specifically targets voxels identified by CSD as containing two or more fascicles. **(A)** Voxel-wise maps of *fvf*_1_. **(B)** Differences between voxel-wise *fvf*_1_ maps.

In Figure 9A, the voxel-wise values of the fiber volume fraction for the larger compartment are displayed. Here, the Fully-Learned Method generally estimated higher *fvf* values compared to the other models, indicating a tendency to estimate higher values than the CSD & MF method.

Figure 9B highlights the differences between the fully analytical CSD & MF model and the two Deep Learning models. The Hybrid Method showed results that were aligned with the CSD & MF model, suggesting its effectiveness in mirroring more traditional analytical approaches. On the other hand, the Fully-Learned Method exhibited a nearly constant bias, diverging from the CSD & MF model to a certain extent.

Overall, these findings from *in vivo* data analysis underscore the practical applicability of the proposed methods, particularly highlighting the nuances in their performance when applied to real-world clinical data as opposed to a controlled synthetic environments.

## 4 Discussion

### 4.1 Performance

Both accelerated methods successfully improved the inference speed of microstructural parameters on both the test set and in *in-vivo* data. This achievement is further highlighted when dealing with voxels containing three fascicles. The acceleration factors become even more impressive due to the inference complexity of the reference method, which increases from $O\left(2N^{2}\right)$ for two-fiber configurations to $O\left(3N^{3}\right)$ for three-fiber configurations. This highlights the potential of the two accelerated methods to handle multiple-fascicles configurations and large dictionary sizes efficiently.

### 4.2 Accuracy

The results from our experiments demonstrate that both the Hybrid Method and the Fully-Learned Method significantly outperform the reference Microstructure Fingerprinting estimation across a variety of simulated scenarios. This superior performance can be attributed to the neural networks' ability to learn and model the complex relationship between the DW-MRI signal and the underlying tissue properties, even in noisy environments. A key factor in this success is the original MF's vulnerability to errors in fascicle orientation estimation, as evidenced in Experiments I and II. The Hybrid Method, which relies on these estimates only in its initial stage, appears to correct for any residual biases in the subsequent stage. In contrast, the Fully-Learned Method, which independently estimates orientations, outperformed CSD in orientation accuracy (as shown in Experiment I, Figure 5) suggesting the potential benefits of benchmarking neural network approaches against established methods in the literature (Canales-Rodŕıguez et al., 2019; Ye et al., 2019; Karimi et al., 2021).

### 4.3 Interpretability

One notable advantage of the Hybrid Method is its interpretability. The method's design allows it to learn useful signal representations more rapidly and with fewer layers. A t-SNE visualization of intermediate outputs through the networks' layers (Supplementary Figure S1) demonstrates that the Hybrid Method effectively captures the *fvf* and *D*_*ex*_ properties after just the first layer, unlike the Fully-Learned Method, which requires more layers to achieve similar structure in its data representations. This rapid learning is likely due to the reliance on the dictionary of fingerprints, which grounds the method in physics-based properties.

### 4.4 Generalizability to diffusion protocol changes

Experiment II focused on the generalizability of the Hybrid Method, revealing that the model trained with the source protocol performed almost as well as the fully retrained model. This finding indicates that the Hybrid Method possesses robust protocol transfer capabilities, suggesting the feasibility of using a single DNN for multiple acquisition protocols. These findings illustrate the Hybrid Method's robust generalization capabilities. The method not only maintains strong performance across different protocols but also shows that a small, yet significant, marginal gain can be achieved by retraining the model on the new protocol. This adaptability is crucial for practical applications, suggesting that the Hybrid Method can be effectively applied in various clinical and research settings with different DW-MRI protocols, and can benefit from fine-tuning to specific acquisition parameters. For the Fully-Learned Method, theoretically, changes in the number or values of shells would necessitate retraining the entire network. However, the use of spherical harmonics implies that changes in gradient directions within a shell should not significantly impact performance.

### 4.5 Generalizability to other microstructural models

The trained models and estimated microstructural parameters in this study were based on a dictionary of presimulated fingerprints obtained with Monte Carlo simulations. This flexible methodology can inherently accommodate other fiber configurations by updating the single-fascicle dictionary, e.g., sampling an undulation, an intra-axonal diffusivity or a dispersion parameter to reflect different brain regions, age, or diseased tissue.

Incorporating more complex fiber configurations at the single-fascicle level such as fanning fibers (Sotiropoulos et al., 2012; Tariq et al., 2016), undulation (Nilsson et al., 2012; Brabec et al., 2020; Rafael-Patino et al., 2020), or glial cells (Taquet et al., 2019) does however require slight modifications of the neural networks. The last layer must match the number of microstructural parameters to estimate and training must be specific to each single-fascicle model.

### 4.6 *In vivo* application

The proposed methods led to massive acceleration of the dMRI data processing, with the Fully-Learned Method performing the estimation of the entire HCP-MGH cohort in 56 minutes, compared to 1714 hours (of single-core equivalent) for the original MF (Table 4). The *in vivo* experiments showed differences between the original MF maps and our proposed methods, with CSD & MF and the Hybrid Method aligning and the Fully-Learned Method predicting higher *fvf* values in specific white matter areas (Figures 8, 9). The stronger similarity between CSD & MF and the Hybrid Method can be explained by the use, in the Hybrid Method, of a signal decomposition in fingerprint space close to the analytical approach of MF. This contributes to the Hybrid Method interpretability, since it is closely aligned with the dictionary approach and its architecture reflects the biophysical organization of axons in white matter (Rensonnet et al., 2021). Additionally, both methods also share a reliance on the same orientation estimation technique. As for the Fully-Learned Method, the consistency of the differences with CSD & MF observed between synthetic and *in vivo* data (Figure 8), combined with its smaller MAEs over CSD & MF in most simulated experiments (Experiments I, II), gives ground to argue for superior accuracy. This suggestion, while promising, deserves further investigation and validation in a broader range of scenarios to fully substantiate its potential advantages over established techniques.

## 5 Conclusion

In this work, we proposed two novel deep learning-based approaches for estimating microstructural features of crossing fascicles in white matter from DW-MRI measurements. The Hybrid Method relies on two stages and uses all diffusion measurements in a voxel along with external estimates of fascicle orientations to determine the properties of each fascicle. In contrast, the Fully-Learned Method leverages a spherical harmonics representation of shells derived from raw diffusion measurements within a voxel. A key distinction between these methods lies in the Hybrid Method's use of a latent space representation through non-negative least squares (NNLS), which allows a high interpretability and protocol transfer. On the other hand, the Fully-Learned Method's employment of a single deep neural network (DNN) contributes to its enhanced accuracy and speedup factor.

Our results demonstrate that both the Hybrid and Fully-Learned Methods yield massive acceleration factors compared to the reference Microstructure Fingerprinting method while outperforming the reference method in estimation accuracy, in various simulated settings. Each method presents its unique advantages and limitations (as summarized in Table 5), highlighting the potential of deep learning in accelerating multi-dictionary matching problems in DW-MRI analysis. Notably, our findings reveal that both proposed methods surpass the reference method in most volume fraction estimations, even when the reference method utilizes ground truth values for fascicle orientations. This underscores the efficacy of deep learning approaches in providing more accurate and efficient solutions for complex interpretation of DW-MRI data, paving the way for their increased application in the field.

**Table 5:** The Hybrid Method is capable of protocol transfer. However, the Hybrid Method is less accurate and depends on the accuracy of an external routine used for orientation estimation.

	**Hybrid Method**	**Fully-Learned Method**
Learning Speed	Fast learning: Requires a small volume of training data.	Slow learning: Requires a large volume of training data.
Inference Speed	Slow inference: The NNLS and the external estimation of fiber orientation reduce the inference time.	Fast inference: The computations of SH coefficients and the inference of the MLP are extremely efficient.
Robustness to missing data	Robust: NNLS can be performed with missing data.	Robust: Missing measurements are interpolated using spherical harmonics.
Protocol transfer capabilities	Capable: No fine-tuning is needed for protocol transfer.	Not capable.
Sensitive to external routine used for orientation estimation	Very sensitive.	Not sensitive since no external routine is used.

Advantages and disadvantages of both fast approaches.

## Data availability statement

The original contributions presented in the study are publicly available. The data and code supporting the findings of this study are available in the GitHub repository of the article at https://github.com/Hyedryn/FastMF_paper/. The repository includes a precalculated dictionary of microstructural features, weights of models trained with PyTorch, and the Python code that encompasses scripts used for voxels simulations, models training, and data analysis.

## Author contributions

QD: Conceptualization, Methodology, Software, Writing – original draft. CF: Software, Validation, Writing – review & editing. BM: Conceptualization, Supervision, Writing – review & editing. GR: Conceptualization, Methodology, Supervision, Writing – review & editing.
