# Supplementary material for: Fast multi-compartment Microstructure Fingerprinting in brain white matter
Source: Front Neurosci. 2024 Jul 19;18:1400499. doi: 10.3389/fnins.2024.1400499 (PMC11294228; doi:10.3389/fnins.2024.1400499)
Supplement: Supplementary file 1 [file Data_Sheet_1.PDF]

Supplementary Material

1 LATENT SPACE VIZUALISATION AND INTERPRETABILITY

As shown in Figure S1, the neural network in the second stage of the Hybrid Method appeared to learn the fvf and D_{ex} properties after just the first layer, while it took the Fully-Learned method three layers to display a similar sample topology.

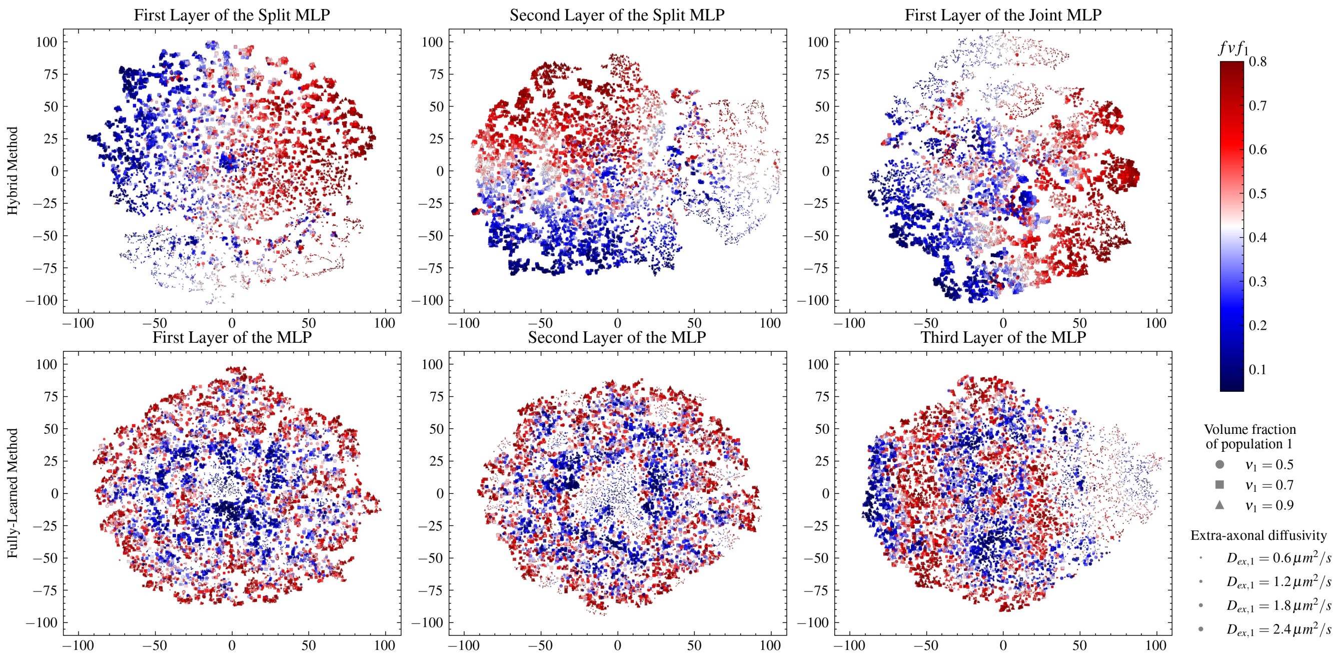

Figure S1: **The neural network of the Hybrid Method learns faster useful signal representations.** Projection in 2-dimensional plane of network activations using t-SNE embedding (Van der Maaten and Hinton, 2008).

2 PERFORMANCE WITH REDUCED GRANULARITY ON MICROSTRUCTURAL PARAMETERS DURING TRAINING

To evaluate the necessity of close-range microstructural parameters during training, the performance of both accelerated methods were investigated when trained with a reduced number of fiber volume fraction (fvf) configurations. Specifically, a network was trained with a dictionary containing 19 equally spaced values within the range $[0.06, 0.78]$ for the fiber volume fraction instead of the original 38 equally spaced values within the range $[0.06, 0.8]$. The extra-axonal diffusivity (D_{ex}) was left unchanged with 10 equally spaced values in the $[0.6, 2.4]$ $\mu m^2\ ms^{-1}$ range. Consequently, the total number of the precomputed fingerprints was reduced to $N = 190$ from the original $N = 380$.

The training process was identical to that used for the original two-fascicle problem, but with the adapted dictionary. The resulting models were evaluated on the test set from Experiment I.

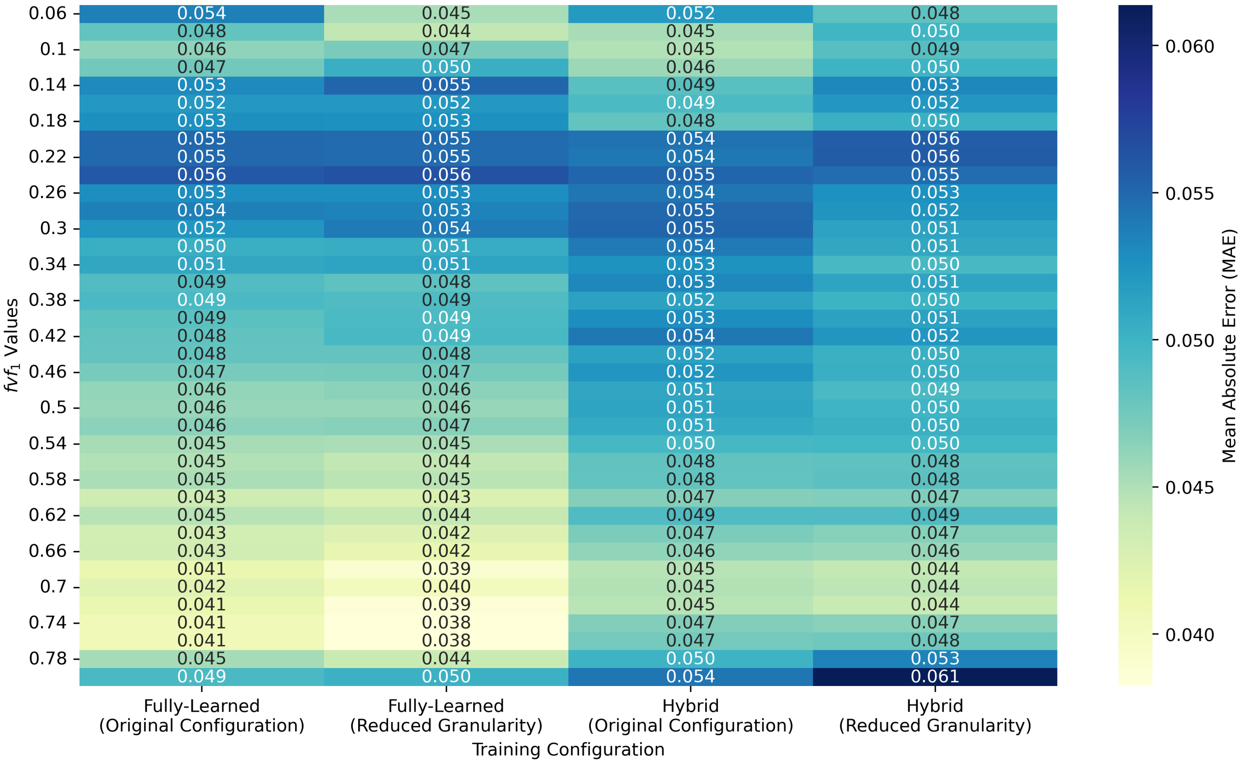

Figure S2: **Consistent performance with minimal deviation were observed, highlighting both methods robustness to reduced granularity in the training set.** Performance comparison of the Hybrid Method and Fully-Learned Method on the test set, trained with a reduced granularity of fvf_1 configurations and trained with the original configuration.

As shown in Figure S2, the performance of both accelerated methods remained consistent despite the reduced granularity in the training set. The mean absolute errors (MAEs) for the fiber volume fractions showed minimal deviation when compared to the models trained on the full training set. The Hybrid Method yielded MAEs of 0.05 for fvf_1 and 0.111 for fvf_2 with both the original model and the model trained with a reduced granularity. The Fully-Learned Method exhibited MAEs of 0.048 for fvf_1 and 0.101 for fvf_2 with the original model and MAEs of 0.047 for fvf_1 and 0.10 for fvf_2 with the model trained with a reduced granularity.

These findings suggest that the models can effectively interpolate within the trained range and maintain reliable performance, reducing the necessity for closely spaced training data points.

3 DETAILS ON TRAINING PARAMETERS AND INFERENCE PERFORMANCES

Table S1. Our neural networks used meta parameters determined by systematic grid-search. Meta parameters and architectural characteristics of both accelerated methods. Note that the number of overall parameters in the network used in the Hybrid Method was lower than that of the Fully-Learned Method, due to its specific split-arm architecture.

	Hybrid Method	Fully-Learned Method
Minibatch size	5000	12 000
Dropout rate	0.1	0.1
Learning rate	3.5×10^{-4}	2×10^{-4}
Training samples	2×10^{6}	2×10^{6}
CASE WITH K=2 FASCICLES:		
Hidden units	2116	2108
Free parameters	5.30×10^{5}	2.11×10^{6}
CASE WITH K=3 FASCICLES:		
Hidden units	2999	2114
Free parameters	8.33×10^{5}	2.12×10^{6}

Table S2. Large speed-up factors were observed for our proposed methods. Detailed computation times for the three methods when performing inference on synthetic data using the MGH Adult Diffusion protocol. The test set was processed on a Skylake Xeon 4116 CPU cluster, without GPU acceleration.

	Microstructure Fingerprinting	Hybrid Method	Fully-Learned Method
CASE WITH K=2 FASCICLES:			
Computation of SH coefficients/voxel	Not applicable	Not applicable	7.8×10^{-4} s
Dictionary rotation time/voxel	8.10×10^{-3} s	8.10×10^{-3} s	Not applicable
Exhaustive fingerprinting time/voxel	9.09×10^{-1} s	Not applicable	Not applicable
Solving NNLS time/voxel	Not applicable	1.47×10^{-2} s	Not applicable
NN forward pass time/voxel	Not applicable	4.24×10^{-5} s	8.12×10^{-5} s
**Total inference time/voxel**	$\mathbf{9.18 \times 10^{-1}}$ **s**	$\mathbf{2.36 \times 10^{-2}}$ **s**	$\mathbf{8.61 \times 10^{-4}}$ **s**
CASE WITH K=3 FASCICLES:			
Computation of SH coefficients/voxel	Not applicable	Not applicable	7.8×10^{-4} s
Dictionary rotation time/voxel	1.03×10^{-2} s	1.03×10^{-2} s	Not applicable
Exhaustive fingerprinting time/voxel	248 s	Not applicable	Not applicable
Solving NNLS time/voxel	Not applicable	1.94×10^{-2} s	Not applicable
NN forward pass time/voxel	Not applicable	6.17×10^{-5} s	1.02×10^{-4} s
**Total inference time/voxel**	**248 s**	$\mathbf{2.97 \times 10^{-2}}$ **s**	$\mathbf{8.82 \times 10^{-4}}$ **s**

REFERENCES

Van der Maaten, L. and Hinton, G. (2008). Visualizing data using t-sne. *Journal of machine learning research* 9
